# Evaluation of the bacteriocin produced by strain 9 lactic acid bacteria isolate for biopreservation

**DOI:** 10.14202/vetworld.2020.2012-2019

**Published:** 2020-09-28

**Authors:** I Dewa Made Sukrama, Juliana Franciska, I Wayan Suardana

**Affiliations:** 1 Department of Clinical Microbiology, Faculty of Medicine, Udayana University, Jl. PB. Sudirman Denpasar-Bali, 80234, Indonesia; 2 Department of Preventive Veterinary Medicine, Graduate Student of Veterinary Medicine, Faculty of Veterinary Medicine, Udayana University. Jl. PB. Sudirman Denpasar-Bali, 80232, Indonesia; 3 Department of Preventive Veterinary Medicine, Laboratory of Veterinary Public Health, Faculty of Veterinary Medicine, Udayana University, Jl. PB. Sudirman Denpasar-Bali, 80232, Indonesia

**Keywords:** bacteriocin, beef, biopreservative, lactic acid bacteria

## Abstract

**Aim:**

This study aimed to determine the effect of the bacteriocin produced by strain 9 lactic acid bacteria (LAB) isolate on the biopreservation of beef.

**Materials and Methods:**

The strain 9 LAB isolate was identified conventionally by culturing with de Man, Rogosa, and Sharpe broth medium followed by Gram staining and catalase testing. The molecular confirmation of the isolate involved analyzing the 16S rRNA gene with specific primers, that is, B27F (5-AGAGTTTGATCCTGGCTCAG-3) and U1492R (5-GGTTACCTTGTTACGACTT-3). Then, the isolate was centrifuged to evaluate the bacteriocin production, and the effect of the biopreservative activity in beef was evaluated by measuring the NH_3_ produced with the Eber test and the organoleptic acceptance from expert panels.

**Results:**

This study confirmed that the strain 9 LAB isolate was a strain of *Pediococcus pentosaceus*, and the bacteriocin product showed biopreservative potential. The biopreservative potential was characterized by a significant decline in the production of NH_3_ and the panel’s acceptance of the texture and tenderness of the beef, compared with the control, after 10 days of constant treatment.

**Conclusion:**

This study highlighted the high biopreservative potency of pediocin produced by *P. pentosaceus* strain 9. This was noted by the production of NH_3_ and the modifications in texture and tenderness.

## Introduction

Lactic acid bacteria (LAB) are a group of Gram-positive, non-sporulating, anaerobic, or facultative aerobic rods or cocci. These organisms are known to produce lactic acid as a major product of carbohydrate fermentation [1]. Furthermore, these organisms are classified into four genera based on the mode of glucose fermentation, temperature range at which they grow, sugar utilization patterns, and cellular morphology. These genera include *Lactobacillus*, *Leuconostoc*, *Pediococcus*, and *Streptococcus*, although the recent use of molecular methods has identified more groups [2,3].

Based on their ability to inhibit the growth of various Gram-negative/-positive bacteria, LAB have thus been adopted as natural starters in food fermentations. These organisms also protect food from spoilage and pathogenic microorganisms through the production of organic acids, hydrogen peroxide, diacetyl, and bacteriocins [4,5].

Specifically, bacteriocins are ribosomally synthesized small proteins known to inhibit spoilage and the growth of pathogenic bacteria in foods [6]. These characteristics have recently been of interest in research due to their possibility of replacing chemical preservation [7]. Furthermore, consumers are currently fascinated by the use of natural food preservation techniques [6,8].

Based on the structural, physicochemical, and molecular properties, bacteriocin peptides from LAB are subdivided into three major classes: Class I, which includes lantibiotics, is small in size (<5 kDa) and contains lanthionine and β-methyllanthionine; Class II, which is also small (<10 kDa) and heat-stable, but non-lanthionine; and Class III, which is characterized by heat-sensitive large molecules [7].

Bacteriocin-producing species have been identified among all the genera of LAB, including *Lactobacillus*, *Carnobacterium*, *Pediococcus*, and *Enterococcus* [6,7]; a large number of these species have been isolated and characterized, and some serve as food preservatives. Some important examples include nisin, diplococcin, acidophilin, bulgarican, helveticins, lactacins, and plantaricins [9].

There have been several studies in Indonesia related to the use of LAB strains as probiotics due to the presence of inherent functional properties that contribute positively toward health. In 2012, Sujaya *et al*. [10] evaluated the application of exopolysaccharides produced by *Lactobacillus* spp. that were isolated from Sumbawa Mare’s Milk as probiotics. Subsequently, Nuraida [11] reported on the effect of some probiotic strains on hypocholesterolemia, immune system stimulation, and the prevention of diarrhea in animals [11]. However, prior studies on the isolation, characterization, and application of bacteriocin are limited. According to a previous study conducted by Suardana in 2015, strain 9 LAB isolate was one of the LAB culture stocks isolated from the gastric juice of Bali cattle. Its activity was evidenced by the killing zones produced in *Staphylococcus aureus* bacterial indicator, at 0.77-1.26 cm, although a negative result was obtained in the treatment with *Escherichia coli*.

This study aimed to determine the effect of the bacteriocin produced by a strain 9 LAB isolate on the biopreservation of beef.

## Materials and Methods

### Ethical approval

The approval from the Institutional Animal Ethics Committee to carry out this study was not required as no invasive technique was used

### Isolate cultivation

The strain 9 LAB isolate was grown in de Man, Rogosa, and Sharpe (MRS) broth medium (Oxoid CM0359.UK Ltd.) under anaerobic conditions with the addition of an Oxoid gas generating kit (Oxoid BR 38) in an anaerobic jar for further incubation at 37°C for 24 h. The isolate was then Gram-stained and underwent a catalase test with the addition of 10 μL of isolate and one drop of 10% H_2_O_2_, before performing molecular confirmation [12].

### Study period and location

The study was conducted from July to September 2016 at the Laboratory of Veterinary Public Health, Faculty of Veterinary Medicine, Udayana University, Denpasar-Bali.

### DNA isolation

The genomic DNA of strain 9 LAB was extracted using a DNA kit (Geneaid, GBB100. Biotech Ltd. Taiwan) according to the manufacturer’s instructions. The culture was centrifuged at 16,000×g for 1 min. The supernatant was then discarded, followed by adding the pellet to 200 μL of Gram-positive buffer, which had previously been filled with lysozyme enzymes (0.8 mg/200 μL). The mixed culture was then incubated at 30°C for 30 min followed by the addition of 20 μL of K proteins and incubation at 60°C for 10 min. Subsequently, 200 μL of GB buffer was added before another round of incubation at 70°C for 10 min. Then, 200 μL of absolute ethanol was introduced and centrifuged at 16,000×g for 2 min. The container tube was replaced, 400 μL of W1 buffer was added to the column, and it was centrifuged at 16,000×g for 30 s. The container tube was swapped with a new tube that had previously contained ethanol; 600 μL of wash buffer was added, and the tube was centrifuged at 16,000×g for 30 s. The column was centrifuged again for 3 min at 16,000×g until a dry column matrix was obtained. At the termination stage, 100 μL of elution buffer (TE buffer) was added and centrifuged at 16,000×g for 30 s. This was performed to dissolve the DNA and capture fragments ready for use [12].

### *16s rRNA* gene analysis

The *16S rRNA* gene of the strain 9 LAB isolate was amplified using B27F universal primers arranged as 5-AGAGTTTGATCCTGGCTCAG-3 and U1492R as 5-GGTTACCTTGTTACGACTT-3, according to the previous procedure, with minimal modifications [12-14]. A total of 1 μL of genomic DNA were introduced to the polymerase chain reaction (PCR) tube, in addition to 25 μL of DreamTaq Green (Thermo Fisher Scientific, K1081.California US), 1 μL each of forward and reverse primers at a concentration of 20 pmol, and sterile water to adjust to a final volume of 40 μL. Therefore, the PCR reaction was programmed using single-step denaturation for 7 min, characterized by 35 cycles of exposure to a denaturation temperature of 94°C for 1 min, an annealing temperature of 56°C for 1 min, and an elongation stage (extension) at 72°C for 1.5 min. Subsequently, the PCR was evaluated using an additional extension temperature of 72°C for 5 min.

### Electrophoresis with 1.5% agarose

The PCR products were analyzed by electrophoresis on 1.5% agarose gel (Sigma 5093-100G) using ethidium bromide staining (Merck, E1510. Germany). This involved the transfer of gel to the electrophoresis tank containing 1× Tris-Borate-EDTA (TBE) buffer, followed by the addition of 5 μL of PCR product and 1 μL of loading buffer, which were then mixed and added to the existing gel wells. Then, electrophoresis was performed at 100 volts for 30 min and viewed under ultraviolet light (transilluminator). The presence of a 1502 bp PCR product indicates a positive outcome [14].

### Sequencing and phylogenetic analysis

The *16S rRNA* gene of the strain 9 LAB isolate was sequenced using a genetic analyzer (ABI Prism 3130 and 3130×l Genetic Analyzer) at the 1^st^ BASE DNA Sequencing, PT (Genetika Science, Jakarta). This procedure involved primers similar to those used for the PCR, and the outcomes were then manually edited to exclude the PCR primer binding sites, using MEGA version 5.2 software (The Pennsylvania State University) This modified result was automatically compared against the sequences of LAB available in databanks (www.ncbi.nlm.nih.gov) using the BLAST program on MEGA version 5.2 sofware. Therefore, the data obtained were subsequently used in phylogenetic tree construction by means of the neighbor-joining algorithm method [15,16].

### Isolation and purification of bacteriocin

Five microliters of the strain 9 LAB isolate were plated on the MRS broth medium (Oxoid CM0359. UK) under anaerobic conditions by adding the Oxoid gas generating kit (Oxoid BR 38. UK) in an anaerobic jar for further incubation at 37°C for 24 h. This mixture was then centrifuged at 12,000×g for 10 min at 4°C and filtered through a 0.22 μm sieve to separate the cell-free supernatants, which was considered the crude bacteriocin. Furthermore, 12 N NaOH was used to adjust the pH to 7.0, and ammonium sulfate (Fluka, Netherlands) was added to obtain 60% saturation, stirred overnight, and subjected to a round of centrifugation at 14,000×g for 1 h at 4°C. Subsequently, the pellets were dissolved with 20 mM potassium phosphate buffer, and the ready semipurified bacteriocin was collected for further evaluation [17,18].

### Application of bacteriocin as a biopreservative

The application of bacteriocin as a biopreservative was evaluated by marinating beef in a bacteriocin solution with a concentration of 500 μL/mL (v/v). This study used 5 g beef samples that were 5×5×2 cm in size and were entirely marinated in 0.85% NaCl solution to align the initial microbial contamination. The samples were divided into three treatment groups as follows: Group I was the control, which was marinated without bacteriocin; Group II was marinated in 500 μL/mL (v/v) bacteriocin for a 5 min treatment; and Group III used similar materials for 10 min. Subsequently, all samples were wrapped in plastic clips and stored in the refrigerator at 5°C, and each treatment was replicated 3 times. Therefore, continuous operation was performed on days 0, 2, 4, 6, 8, and 10. The change in beef quality among the treatments in each observation was measured according to the meat quality parameters in this study.

### Meat quality parameters

The meat quality parameters observed included spoilage, which was evaluated with the Eber test, and the texture and tenderness, which were evaluated by expert panels. The Eber test comprises a combination of 1 part 37% HCl (Merck 1.00317.2500.Germany) in three parts 96% alcohol (BD.SKU74000CPC) and 1 part 74.12 g/mol ether (Merck 1009301000.Germany). The results were declared negative in the absence of any white clouds around the meat, positive 1 (+) if a small quantity of white clouds formed (after 10 min), positive 2 (++) if quite a lot of white clouds formed (within 5-10 min), and positive 3 (+++) if large white clouds formed (within <5 min). Based on the formation of NH_4_Cl with the Eber test, 0 is indicative of a negative (-) outcome, whereas 1 (+), 2 (++), and 3 (+++) denote positive outcomes.

Furthermore, the texture and tenderness were evaluated by five trained panelists (female students in their eighth semester) from the Faculty of Veterinary Medicine; the results were scored as 3, 2, and 1 for fresh, mushy, and watery meat, respectively. These values were then summed, and an average calculated, which was subsequently statistically analyzed with a one-way ANOVA, followed by Duncan’s multiple range test. Next, a relationship between treatments and storage period was identified based on the formation of NH_4_Cl gas and the texture and tenderness, and the results were further subjected to a regression correlation analysis using SPSS 18 software.

## Results

### Isolate cultivation

The strain 9 LAB isolate cultivation was characterized by luxuriant growth on the MRS broth medium in an anaerobic atmosphere, Gram-positive results, and a negative catalase test. The results of this study confirmed the strain 9 isolate to be an LAB, so we could proceed to the next analysis.

### *16S rRNA* gene analysis

**Figure-1:**
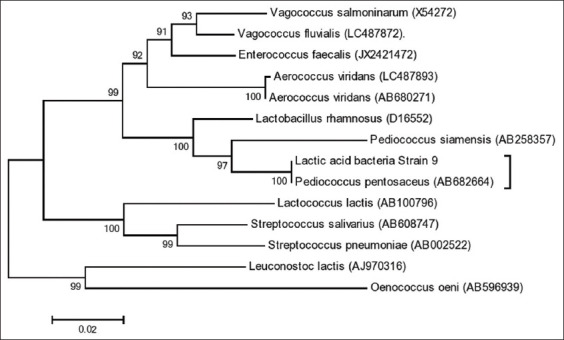
Phylogenetic tree constructed using the neighbor-joining algorithm of 16S rRNA gene nucleotides sequence. The number in each phylogram branch indicates a bootsrap value (%) by 1000-replication multiple, and scale shows two per 100 nucleotide substitutions.

The *16S rRNA* gene of the strain 9 LAB isolate was amplified successfully using the B27F and U1492R primers previously characterized by the clear 1502 bp PCR product. Therefore, the sequencing result was aligned against several LAB data from GenBank, which identified the bacteria as *Pediococcus pentosaceus*, of the group *P. pentosaceus* (AB682664) with a bootstrap value of 100 (Figure-1).

The assembly of the strain 9 LAB isolate with *P. pentosaceus* (AB682664) in one group was confirmed by the data in Table-1, which shows the similarity in the nucleotide sequences of both strains. However, the intrinsic characteristics are quite different from *Oenococcus oeni* (AB596939), with 164 different nucleotides per 1000 alignment. Based on this comparison concept, samples with nucleotide similarities >90% or differences between 1 and 1.5% (14-22 bp) are categorized as the same species [13,14,19].

**Table-1:** Pyelogram of strain 9 LAB isolate against other samples in GenBank. The phyelogram was constructed based on neighbor-joining algorithm.

	Lactic acid bacteria strain 9	*Aerococcus viridans* (LC487893)	*Aerococcus viridans* (AB680271)	*Enterococcus faecalis* (JX2421472)	*Lactobacillus rhamnosus* (D16552)	*Lactococcus lactis* (AB100796)	*Oenococcus oeni* (AB596939)	*Pediococcus siamensis* (AB258357)	*Pediococcus pentosaceus* (AB682664)	*Streptococcus* *salivarius* (AB608747)	*Streptococcus pneumoniae* (AB002522)	*Vagococcus salmoninarum* (X54272)	*Vagococcus fluvialis* (LC487872)
Lactic acid bacteria strain 9													
*Aerococcus viridans* (LC487893)	0.093												
*Aerococcus viridans* (AB680271)	0.092	0.001											
*Enterococcus faecalis* (JX2421472)	0.085	0.060	0.059										
*Lactobacillus rhamnosus* (D16552)	0.043	0.072	0.071	0.066									
*Lactococcus* *lactis* (AB100796)	0.134	0.133	0.131	0.118	0.137								
*Leuconostoc lactis* (AJ970316)	0.132	0.136	0.134	0.120	0.130	0.156							
*Oenococcus oeni* (AB596939)	0.164	0.177	0.175	0.172	0.171	0.162	0.124						
*Pediococcus siamensis* (AB258357)	0.055	0.114	0.113	0.099	0.067	0.165	0.160	0.191					
*Pediococcus pentosaceus* (AB682664)	0.000	0.093	0.092	0.085	0.043	0.134	0.132	0.164	0.055				
*Streptococcus salivarius* (AB608747)	0.126	0.127	0.125	0.101	0.119	0.072	0.152	0.165	0.158	0.126			
*Streptococcus pneumoniae* (AB002522)	0.136	0.124	0.122	0.115	0.128	0.087	0.146	0.149	0.167	0.136	0.044		
*Vagococcus salmoninarum* (X54272)	0.092	0.066	0.064	0.046	0.080	0.114	0.146	0.188	0.107	0.092	0.112	0.122	
*Vagococcus fluvialis* (LC487872)	0.078	0.059	0.058	0.034	0.070	0.114	0.131	0.180	0.095	0.078	0.108	0.117	0.030

### Application of bacteriocin as a biopreservative

The Eber test is one of the methods used to determine the spoilage of meat and its derivatives and is shown by the production of NH_3,_ which is produced from the biochemical activity of detrimental microorganisms. The gas released is recognized by the formation of white clouds through a reaction between NH_3_ and HCl around the test sample.

The Eber test showed the release of NH_3_ from the 6^th^ to the 10^th^ day of observing the control group and the groups treated with bacteriocin and marinated for 5 and 10 min. The production of NH_3_ was reduced in the treatment groups based on the relatively smaller amount of white clouds formed, and the production time was also longer. Furthermore, the data were converted into 0 for negative results (−), 1 for (+), 2 for (++), and 3 for (+++), after being transformed by √ 0.5.

**Figure-2:**
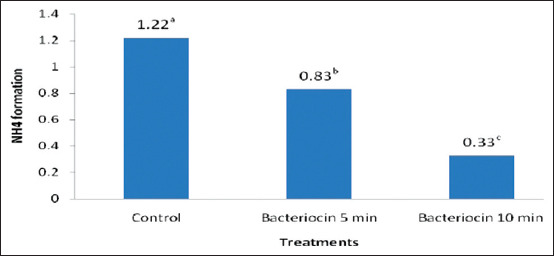
The properties of NH_4_ produced after the reaction of NH_3_ released during meat spoilage and the HCl substrate from Eber test between control and treatment groups. The table was construct base on Duncan’s multiple range test, after convertion to √0.5.

**Figure-3:**
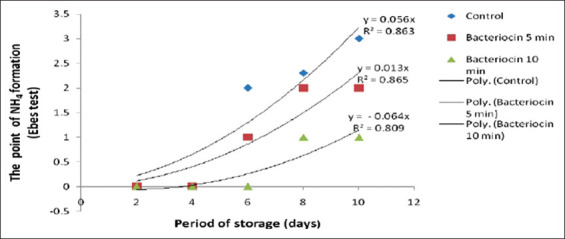
Quadratic curves of NH_4_ gas formation on the Eber test, resulting from the interaction between NH_3_ released during spoilage and HCl substrate, for beef immersed in bacteriocin for 5 and 10 minutes, and the control.

Figure-2 shows that there was a significant difference (p<0.05) in the amount of NH_4_ formed in the group treated for 5 min compared with the control and with the group treated for a duration of 10 min. Furthermore, the regression analyses of the storage period for NH_4_ gas formation for the control and 5 and 10 min bacteriocin treatments in Figure-3 showed regression curves of Y=0.056x (correlation coefficient [R^2^]=0.863); Y = 0.013x (R^2^=0.865); and Y=−0.064x (R^2^=0.809), respectively.

Figure-3 shows a strong correlation between the storage period and the formation of NH_4_ gas. This is evidenced by the increase in the production of NH_4_Cl by the control compared with the 5 and 10 min bacteriocin treatments. In addition, the graphics indicate a significant correlation between the production of NH_4_Cl and the storage period.

**Figure-4:**
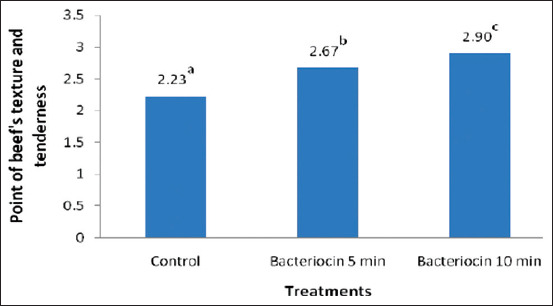
The properties of beef’s texture and tenderness between control and the treatments with bacteriocin. The table was construct base on Duncan’s multiple range test after conversion of descriptive data.

Moreover, the organoleptic criteria of texture and tenderness were used to determine meat quality, and the result was congruent with the Eber test. These parameters are strongly associated with the amount of muscular connective tissues and have been selected as the most important qualitative attributes by the average consumer [20,21]. In addition, texture and tenderness, which were classified as fresh, mushy, and watery meat in this study, were closely associated with the spoilage process. Figure-4 shows the results of the panel tests on the effect of beef immersed in bacteriocin for 5 and 10 min compared with the control on the properties of beef’s texture and tenderness.

**Figure-5:**
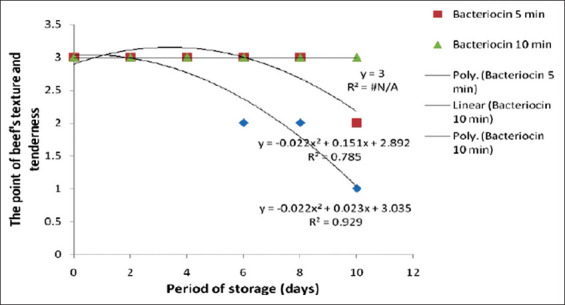
Quadratic curves of beef texture and tenderness between the sample immersed in bacteriocin and the control on storage.

Figure-4 indicates that there was a significant difference (p<0.05) between the control experiment and the beef immersed in bacteriocin for 5 and 10 min. Figure-5 shows the regression correlation analysis, which displays a close correlation between the period of beef immersion and the storage phase. Furthermore, the results on texture and tenderness for the control and treatment with bacteriocin for 5 and 10 min were expressed with regression curves of Y=3 (R^2^=N/A); Y=−0.22X^2^+0.151X+2.892 (R^2^=0.785); and Y=−0.022X^2^+0.023X+3.035 (R^2^=0.929), respectively.

Figure-5 shows higher scores for texture and tenderness in the beef treated with bacteriocin than the control, which showed a resilient decline up to day 10 of observation.

## Discussion

The low carbohydrate content of beef (1% or less) indicates a higher tendency for spoilage by organisms that utilize free amino acid content as a source of energy. Furthermore, the process leads to the production of ammonia, hydrogen sulfide, indole, skatole, and amines [22], although storage at refrigerator temperatures significantly reduces the growth rate of all genera. However, some organisms are resistant in this regulated environment and have been identified in fresh meats. These include *Micrococcus, Streptococcus*, *Escherichia*, and *Klebsiella* which may be eliminated in samples treated with antibiotics or other inhibitors [22].

The strain 9 LAB isolate and the bacteriocin produced in this research were confirmed to be *P. pentosaceus* and pediocin, respectively. Based on different sources, there has been a general increase in the application of bacteriocin-producing strains in the health and food industry [23]. This is evidenced by several related studies, including the study by Cortés *et al*. [24] which showed maximum bacteriocin production by *P. pentosaceus* at 37°C that occurred predominantly at the end of the exponential growth phase, followed by a stabilized yield rate, during the 24 h incubation period. Therefore, pediocin was confirmed to have potential use as a food biopreservative.

Other studies have identified pediocin A as a bacteriocin produced by *P. pentosaceus*; it is grouped as a Class II bacteriocin, with a molecular weight of 2.7 kDa. This product was generally sensitive to proteolytic enzymes and heat (10 min at 100°C). Meanwhile, pediocin F, which is produced by *Pediococcus acidilactici* and is placed in a similar category, has a weight of 4.5 kDa and is known to be sensitive toward proteolytic enzymes, is heat and organic solvent resistant, and is active under a wide range of pH levels [6]. Several forms of pediocin-producing *Pediococcus* spp. have also been isolated and evaluated, and despite their structural similarities, the molecular weights always varied, alongside their spectrum of antimicrobial activity. Furthermore, the bactericidal activity against Gram-positive food spoiling and pathogenic bacteria increases the product importance in the class of biopreservatives [25].

Meat and its products have been considered excellent growth media for a variety of microflora (bacteria, yeasts, and molds), including some pathogens. This phenomenon makes the intestinal tract and the skin of animals a major source of microorganisms, although the microflora composition depends on various factors: (a) Preslaughter husbandry practices (free range vs. intensive rearing); (b) age of the animal at the time of slaughtering; (c) handling during slaughtering, evisceration, and processing; (d) temperature controls during slaughtering, processing, and distribution; (e) preservation methods; (f) type of packaging; and (g) handling and storage by the consumer [26]. In addition, the pediocin produced by the strain 9 LAB isolate was proven to be a biopreservative, which was indicated by its abilities to inhibit the formation of NH_4_Cl in the Eber test on meat treated with pediocin. This assumption is supported by Lawrie’s theory [21], which states that the process of decay caused by spoilage organisms in meat results from the use of free amino acids as an additional energy source. The reactions produce hydrogen, carbon dioxide, and ammonia. Furthermore, reduced NH_4_ formation in beef treated with bacteriocin occurs due to reduced NH_3_ release, which is a result of the antibacterial activity of pediocin killing many of the spoilage microorganisms. In general, pediocin inhibits bacteria through three mechanisms: (1) Creating work pores in the cytoplasm membrane of cell targets, (2) destroying the intracellular pH of the bacteria target, and (3) inhibiting the proton motive force for energy production in the bacterial cell. Pediocin was linked to cell targets through active sites such as the negative charges of phospholipid groups in the cell wall. The previous studies have shown a relationship between the amount of lipoteichoic acid in the cell wall of Gram-positive bacteria (G+) and increased pediocin activity against G+ bacteria. There is higher pediocin activity when the acid concentration is increased in the walls of bacterial cells [27,28]. This result agrees with the previous statement, in which pediocin was evaluated as low-molecular-weight (2.7-17 KDa) cationic molecules. Pediocin is composed of a hydrophilic N-terminal part, which contains the pediocin box (YGNGV) motif, and a hydrophobic or amphiphilic C-terminal variable part, often produced by some *Pediococcus* bacteria. Furthermore, pediocin has inhibition properties against several sensitive bacterial cells, resulting from the ability to act on the cytoplasmic membrane during pore formation and through the absorption of amino acids in the phospholipids of the target cell cytoplasmic membrane [29]. Its effectivity of inhibiting food spoilage has also been identified against some species of *Enterococcus*, *Listeria*, *Leuconostoc*, and *Staphylococcus* [30].

Moreover, the previous reports have defined meat spoilage as a change in quality, leading to undesirable characteristics, making it unfit for consumption. This includes the development of off-flavors, off-odors, and often the formation of slime, resulting from the breakdown of valuable contents (fat, protein, and carbohydrates). In addition, microbial spoilage causes sour tasting, discoloration, gas production, pH changes, slime formation, degradation of structural components, and appearance modification [31]. The most important qualitative attributes of eating are texture and tenderness, which basically depend on three main components: (1) The degree of sarcomere contraction or “length,” (2) the extent of structural myofibrillar protein integrity/degradation (proteolysis), and (3) the connective tissue content/composition [32]. Conversely, some strains of the genera *Pseudomonas* and *Achromobacter* grow at refrigerator temperatures and are often able to degrade gelatin, with poor decomposition activity against natural proteins, including casein and purified myofibrillar proteins. However, the physical changes produced tend to be more apparent compared with the chemical effects, as observed in terms of color, odor, flavor, and tenderness [33]. Moreover, there is a high tendency to apply bacteriocin-producing LAB strains in biopreservation, evidenced by the higher texture and tenderness scores for meat immersed in pediocin than the control after 10 days. This outcome confirmed the spoilage inhibition characteristics of bacteriocin, which agrees with findings from a study by de Costa [34], who used LAB as a biological preservative on meat. Thus, using LAB are an important approach to maintain the microbiological quality and safety of meat and its products to extend food shelf-life and foster consumer acceptance. One such method involves using bacteriocin as a biopreservative, such as pediocin-producing *P. pentosaceus* strain 9.

## Conclusion

The biopreservative potency of pediocin produced by *P. pentosaceus* strain 9 on beef is confirmed to be feasible. This outcome is evidenced by the production of NH_3_ and the evaluation of texture and tenderness. There is a need for subsequent studies to perform a more complete test on the aspect of potency.

## Authors’ Contributions

IDMS, JF, and IWS created and designed the experiment. JF and IWS performed PCR. IDMS, JF, and IWS drafted and revised the manuscript. All authors have read and approved the final manuscript.
